# What Do Scientists Mean When They Talk About Research Animals “Volunteering”?

**DOI:** 10.1163/15685306-bja10139

**Published:** 2023-08-11

**Authors:** Alexandra Palmer, Beth Greenhough, Pru Hobson-West, Gail Davies, Reuben Message

**Affiliations:** School of Biological Sciences, University of Auckland, Auckland, New Zealand; School of Geography and the Environment, Oxford University Centre for the Environment, University of Oxford, Oxford, UK; School of Sociology and Social Policy, University of Nottingham, Nottingham, UK; Department of Geography, University of Exeter, Exeter, UK; Science, Technology and Innovation Studies, School of Social and Political Science, University of Edinburgh, Edinburgh, UK

**Keywords:** agency, animal research, animal behavior, ethics, laboratory, animal psychology, voluntary, welfare

## Abstract

This paper examines discourses around “volunteering” in animal research. Through a qualitative textual analysis of the scientific literature using animals in behavioral and psychological research, we demonstrate that “voluntary” and related terms are used by scientists in a variety of distinct ways, which carry a range of ethical and political connotations. While any reference to volunteering might be assumed to imply free, unconstrained, and unpaid participation in an activity, in the animal research literature the term is often used simply to signal a lack of physical restraint, even though other human-imposed constraints are at play. Though truly voluntary behavior may be impossible, we nevertheless argue that there is a case for seeing use of the language of volunteering as an ethical or political move in which scientists aim to highlight a goal of minimizing human control, promoting animal welfare, or representing their research as ethically acceptable.

Imagine a cognitive science laboratory study in which group-living monkeys are given free access to a research cubicle in which they are presented with puzzles; the monkeys are free to leave the cubicle at any time. Research along these lines is very often described as “voluntary” for the monkeys. Now imagine the monkeys are presented with food rewards for correct answers to puzzles; is their participation voluntary in this case? What about if, rather than a puzzle, the monkeys undergo something unpleasant, like electric shocks, in return for food rewards, or are on a restricted food regime unless they participate? Is this still voluntary? How would we describe these studies if the subjects were human?

These questions illustrate the central purpose of this paper, which is to unpack the discourse of nonhuman-animal (hereafter, animal) “volunteering” in the context of scientific research. Although the scenarios presented above are hypothetical, they illustrate a real situation: that the use of the term “voluntary” is quite variable in animal research. Our goal is not only to describe the contexts in which the language of volunteering is used in animal research, but also to explore what discourses of volunteering do politically and ethically, and how these discourses construct the relationship between researchers and the animals they study. Our analysis of volunteering discourses sits within a long tradition of using discourse and rhetoric analysis to examine language used in scientific communications (e.g., Bazerman, 1984, 1988; Collins et al., 2018; Gilbert & Mulkay, 1984; Latour & Woolgar, 2013; McLeod et al., 2019) and representations of animals in scientific contexts, such as use of language related to agency (Birke & Smith, 1995; Cook & Sealey, 2017; Durham & Merskin, 2009). Such analyses are premised on the idea that language is important for shaping perceptions of the subject matter, and can therefore reveal implicit meanings in texts and show how texts are constructed to persuade readers. This research additionally contributes to ongoing discussions about the relationship between animal agency and human control (e.g., Despret, 2013; Howell, 2018; Pearson, 2015), the implications of viewing research animals as agentive subjects − as “workers” rather than as “lab tools” (Clark, 2014; Haraway, 2008) − and the meaning of “consent” for animals, in research and other settings (Arnason, 2020; Ashall et al., 2018; Fenton, 2014; Greenhough & Roe, 2011; Healey & Pepper, 2020).

We begin by reviewing how the language of volunteering is used in animal research, based on qualitative textual analysis of the published scientific literature. We then explore how the language of volunteering is employed to describe experiments that accord animals differing levels of agency and employ different kinds of incentive. In each case we examine the conceptual debates around animal agency, relational autonomy, and the limits to notions of informed consent, which can be used to explore the implications and limitations of using voluntariness as a way of describing specific experimental practices in animal research. We demonstrate that although reference to volunteering in humans generally indicates unconstrained and unpaid participation, in animal research literature the term is often used simply to signal a lack of physical restraint, even though other human-imposed constraints are used. We conclude by considering the implications of using the language of volunteering in animal research, arguing that while “voluntary” cannot be considered a technical term with a consistent meaning, in some cases it can be viewed as an ethical or political move in which researchers aim to highlight a goal of minimizing human control, promoting animal welfare, or representing their research as ethically acceptable. Nevertheless, efforts by some animal behavioral researchers to make animals’ participation *as voluntary as possible* remains a worthy aspiration.

## How the Language of Volunteering is Used in Animal Research

To explore how the term “voluntary” is currently used in animal research, we collected a sample of recent scientific publications that use “voluntary” or related terms with reference to research animals. Using Google Scholar, we performed searches for the term “voluntary” in publications also containing the phrases “animal behavior” (both U.S. and U.K. spellings), “laboratory animal,” or “zoo,” to ensure we obtained examples from a variety of settings. To make this task manageable, we restricted our search to texts published in 2018. This search yielded 75 publications and conference abstracts, of which 55 contained relevant uses of the term “voluntary.” We discarded results in which the term was applied to humans or featured only in the reference list. Based on this initial search, we then collected a further 83 publications via snowballing and searches for related terms such as “willing.” This approach provided us with a substantial, if not representative, sample of relevant animal research literature.

We assigned each paper an overarching code reflecting how the language of volunteering was used (e.g. “voluntary exercise,” “voluntary ingestion (food)”). Based on this qualitative analysis, we merged and renamed codes as appropriate to produce a list of the nine most common uses, outlined in Table 1. We found that most commonly, the language of volunteering was used in behavioral and psychological research with specific aims, such as investigating motivations for human behaviors including alcoholism and exercise, exploring animal cognition and behavior, or attending to animal welfare. However, the exact meaning of the term “voluntary” was rarely explained, nor could we find existing work suggesting how “voluntary” ought to be defined in animal research.

We therefore elected to explore discourses of volunteering in the animal research literature as represented in our sample. By *discourse,* we refer not just to language itself, but to how language “encodes, reflects, (re)produces, and challenges social norms” (Paterson & Gregory, 2019, p. 27). In particular, we focus on how these discourses relate to two common ideas: that volunteering means doing something (a) willingly as a free agent, and (b) without being forced or paid. These ideas are both signaled in the Cambridge Dictionary’s (2020) definition of “voluntary” as an adjective describing an activity “done, made, or given willingly, without being forced or paid to do it.” For each of these ideas, we first outline how they have been approached in the social sciences and humanities before exploring how these ideas are reflected (or not) in the animal research literature, drawing on our sample of research publications as well as grey literature from a specific U.K.-based debate about neuroscience research involving primates.

## Willingness and Agency

### Background: Agency and Constraint

Are (at least some) animals “agents?” If so, what is meant by “agency?” Responses to these complex questions have come from a variety of disciplines (McFarland & Hediger, 2009). Our premise is that at least some animals can be agents, in the sense of being independent creatures with their own preferences and desires who are capable of shaping the world through their actions. We are not alone in making this assumption; this view lies at the heart of the so-called “animal turn” within many social science and humanities disciplines (Haraway, 2008; Ritvo, 2007). Our concern in this section is the relationship between *agency and constraint* in the animal research laboratory. After briefly reviewing key ideas about agency and constraint in the humanities and social sciences, we consider how the context of the laboratory might constrain animal agency, specifically via processes of training and breeding.

Despite acknowledging animal agency, animal studies scholars have observed that animals are still constrained by biopolitical control strategies implemented by humans, and that humans often hold more power than animals in cross-species relationships (Braverman, 2015; Srinivasan, 2013). The ways in which animals confound and resist human control have become significant areas of research in social science (e.g., Hodgetts & Lorimer, 2020), history (Howell, 2018; Pearson, 2015), and philosophy, where scholars have explored the meaning of animal “dissent” (Arnason, 2020; Fenton, 2014; Healey & Pepper, 2020). In this literature, questions remain about how to recognize animal dissent, and − even more challenging − how to define and recognize its opposites: “consent,” “assent,” or “voluntary” behavior (see also Despret, 2004, 2013; Gray et al., 2018, Greenhough & Roe, 2011).

One obstacle to developing universal definitions of these concepts is that they are context-dependent, since choices and expressions of agency are inevitably constrained and influenced by external factors (Healey & Pepper, 2020). As summarized by Gruen (2011), for both humans and animals “[t]o act autonomously does not require being completely free from constraints” (p. 149) since one can still determine how to act considering social factors that not only restrict available choices but also shape personal desires and interests. This is the concept of “relational autonomy” highlighted by feminist philosophers (see Mackenzie & Stoljar, 2000). This means it is impossible to separate individual agency and constraint completely, and we can only talk about personal autonomy as taking place within a social context (see also Despret, 2013).

This social context might be deliberately shaped by powerful parties with the goal of pushing others towards certain choices, potentially involving an element of deception. Governments may use behavioral science techniques to direct people toward specific kinds of actions without outlawing undesirable behavior, a form of neoliberal governmentality (Lorenzini, 2018). Recent work on concepts of “nudge” and “neuroliberalism” highlight efforts by governments to subtly structure people’s choices to lead them the “right” decisions, such as making healthy lifestyle choices (Baldwin, 2014; Jones et al., 2014; Whitehead et al., 2019). Similar strategies may be applied to animals. For example, Holloway (2007) demonstrates that despite claims that Automatic Milking Systems (AMS) used on dairy farms allow cows to be milked “voluntarily” − a term used explicitly by some farmers cited by Holloway, and in one research paper we surveyed on “voluntary cow traffic” toward AMS (Wildridge et al., 2018) − in practice, AMS systems are installed to push cows to make “the right choices and exercise their freedom appropriately.” For example, some AMS systems are installed in a one-way system, such that cows must be milked to access food and water, and cows may be bred for their suitability for AMS systems. Thus, “autonomy granted to cows under AMS is rhetorical rather than ‘real’” (Wildridge et al., 2018, p. 1050). A similar argument could be made about some animal research described as voluntary.

### Application to Animal Research: Training and Breeding

Applying these ideas to animal research encourages us to think about how research animals’ choices are shaped and constrained by the circumstances established by human researchers. This may first happen via training. Very often in the animal research literature we surveyed, training was viewed as compatible with volunteering; indeed, training was sometimes cast as *enabling* choice and as a fundamental element of “voluntary” research with animals (see also Arnason, 2020). For example, in a paper discussing efforts to have laboratory-housed chimpanzees voluntarily move between enclosures (Table 1), Bloomsmith et al. (1998) stated that “[p]ositive reinforcement allows increased choice as the animals volunteer to participate or not” (p. 339). One might liken this to a situation in which policymakers view neuroliberal strategies as still compatible with people’s ability to make free choices. However, we encountered one study where voluntary behavior was cast as antithetical to training: Chertoff et al. (2018) described a study in which “[p]articipation by the gorillas was voluntary in that no training or reinforcement was provided” (p. 292), implying that there may be some disagreement about the role of training in voluntary behavior among animal researchers.

Debates about the relationship between training and voluntary behavior came to the fore in discussions around a report undertaken in the U.K. by the Animal Procedures Committee (2013) on the experience of nonhuman primates (hereafter, primates) in neuroscience research, commonly referred to as the Pickard Report. During visits by members of the working group to research establishments, research staff raised the idea that primates “may actually look forward to performing the experimental tasks” (p. 102). In contrast, several animal protection groups argued that given evidence in the report that primates are kept hungry and thirsty as motivation for performing tasks, and force may still be used in training (e.g., use of collar and pole training), researchers interviewed for the report make a “preposterous misinterpretation” by viewing “a monkey’s desperate thirst or hunger as ‘willingness’ to sit in a ‘training chair’” (Animal Aid et al., 2014, p. 4). The Animals in Science Committee (2014) also challenged the Pickard Report’s conclusion, noting that because primates “perform tasks for so long (years in many cases) they could be performing habitually” (p. 3). In other words, the training of primates used in neuroscience research might account for their participation and should not be misread as active “willingness.” Similarly, Fenton (2014) argues that when chimpanzees used in research display “learned helplessness,” it is no longer morally significant whether they appear to dissent or comply with harmful research.

Another interesting practice mentioned in the Pickard Report is collaboration between scientists and breeding colony managers to select “well-motivated subject[s]” (p. 100). This hints at how humans might shape research animals’ preferences and behavior before they are even born, via breeding, like the cows Holloway describes in AMS systems. Like training, the use of breeding to shape research animals’ preferences is generally presented as compatible with volunteering. For example, mice used in exercise studies (Table 1) may be “selectively bred for high voluntary wheel-running” (Kelly & Pomp, 2013, p. 349); the same is true in some rodent studies examining alcohol and drug consumption (Table 1; e.g., Chester et al., 2004). Here, one may argue that humans have played an even more significant role in shaping the behaviors an animal is inclined to perform. The behavior of domesticated animals could, in a sense, be seen in the same way, though less so if one views domestication as more of a process of mutualism or co-evolution (Cassidy and Mullin, 2007). Still, animals with certain personalities and life histories more readily engage in research activities (Herrelko et al., 2012; Morton et al., 2013), and humans may have played a significant role in shaping personality and life history via breeding and training.

## The Use of Force and Rewards

### Background: Coercion and Altruism

In the previous section, we discussed how humans might establish structures that shape the preferences of animals before the research even takes place, namely via breeding and training. We now consider the idea that voluntary behavior cannot involve any force or payment, which in turn relates to the assumption that voluntary behavior in humans should be driven by “morally worthy” motivations like altruism. Though the principle of freely given, autonomous, informed consent dominates in discussions of human research participation in clinical trials, social science research has demonstrated that in practice, people may feel compelled to participate in research due to broader contextual factors. For example, Rajan (2007) describes how the recruitment of unemployed mill workers from Mumbai’s former industrial district into paid clinical trials can hardly be considered voluntary in the context of few other sources of employment or healthcare. Similarly, Fisher and Walker (2019) point out that phase one clinical trials with healthy volunteers in the U.S. are very often run in a manner that attracts vulnerable individuals, some of whom view the money as “too good to refuse” (p. 5). For some, volunteering for clinical trials can even become a “job,” yet because clinical trial “volunteering” is not framed as work, they lack protections afforded to workers. For these reasons, financial remuneration of human volunteers is often frowned upon if it is viewed as potentially causing “coercion” or “undue inducement” (NHS Health Research Authority, 2014).

We see in this brief example two important points. First is the idea that sociopolitical contexts can constrain the available choices and push people toward “volunteering” for something they would not otherwise do. Second, financial incentives can transform volunteering into something that would be more commonly described as “work,” which, unlike volunteering, is commonly defined as a paid activity (e.g., Cambridge Dictionary, 2020b). This distinction reflects an underlying concern about whether human volunteers must be involved for the “right reasons,” i.e., motivated by altruism or personal self-fulfillment rather than money (e.g., Roe & Greenhough, 2018). For humans, the motivation and nature of rewards or payment are therefore considered important in determining whether certain activities are “voluntary”: money should not be the reward volunteers are looking for (even if, in reality, some are motivated by money), but something more intangible, like a contribution to economic (Fortun, 2008) or scientific (Hoeyer, 2003) progress. Therefore, to “volunteer” arguably carries a connotation of action that is motivated by more “morally worthy” reasons than work.

### Application to Animal Research: Alternative Options and Food Rewards

Applying these ideas to animal research encourages us to think not only about whether the animal has the option of doing or avoiding an activity, but also what other options are available for those who opt out. A good example of this is illustrated in cognitive research conducted by Gazes et al. (2013), in which the researchers compared the test results of rhesus macaques housed in a laboratory with those housed in a free-ranging field station. In both settings, macaques were given free access to the test apparatus, and though they received food rewards for correct answers, they were also not deprived of food; as such, the researchers describe participation in the cognitive tests as “voluntary.” However, although all the laboratory-housed subjects participated in the tests, only 51% of non-infant field station monkeys participated at all. This result suggests that caged animals are more likely to “volunteer” to participate in research than free-ranging animals. Given that they did not choose to live in a constrained environment, it is reasonable to ask whether captive animals can ever participate in research voluntarily. In other words, as in the case of human participation in clinical trials, certain contexts and environments can push animals toward “volunteering” in research, such as food deprivation (see previous discussion of the Pickard Report) or captivity.

To give another concrete example, food is often described as a “reward” in the context of animal research, “voluntary” and otherwise; we might therefore think of food as equivalent to financial payment for humans. However, as research by Frans de Waal (2005) illustrates, quantity is not everything, since capuchins who previously appeared very happy to receive cucumbers became upset when their peers received grapes, once again demonstrating the contextual nature of individual decisions (in this case, a perceived inequality). Thus, as with human decisions to volunteer in clinical trials, animals’ decisions must be understood not only in terms of the quantity of payment offered, but also in terms of the broader sociopolitical context (Greenhough, 2007).

In our analysis of the animal research literature, we noticed animal behavior commonly referred to as simultaneously “reward based and voluntary” (Goodrowe, 2003; Margulis, 2017; Wondrak et al., 2018). Again, Chertoff et al. (2018) present an exception to this trend when they describe gorillas’ participation as voluntary *because* no training and reinforcement (i.e., food rewards) were provided. People commonly associate volunteering with performing an action for a worthy cause (e.g., altruism). In our analysis, Chertoff et al. (2018) come closest to including such normative commitments in their definition of volunteering. However, though we might acknowledge that (some) animals can be motivated by altruism (de Waal, 2011), it is unclear how humans would attempt to detect this without veering into “altruistic anthropomorphism” in contexts where animals’ wellbeing is compromised for an abstract “greater good” (Ashall, 2017). Furthermore, communicating to animals the intentions behind research is likely impossible. This means that even if animals possess capacities for altruism, we cannot accurately convey to them how others will benefit from the research (Arnason, 2020; Fenton, 2014; Healey & Pepper, 2020).

If we cannot reasonably expect animals’ participation in research to be motivated by altruism or scientific contribution, then what motivations should we find acceptable to consider animals’ participation “truly” voluntary? One route forward could be to utilize the distinction between “extrinsic” and “intrinsic” reinforcement, the former referring to behaviors that are performed to obtain an external reward like food, and the latter to behaviors that are intrinsically rewarding, such as play, hunting, and exploration (Tarou & Bashaw, 2007). Though it could be tempting to classify the latter as more desirable than the former, it should be noted that not all intrinsically reinforced behaviors are considered desirable − repetitive, stereotypic behaviors being one example − and “rewards” might be widely considered desirable, like human contact, toys, sensory stimulation, or care and protection as in cases of companion animals (Coulter, 2016). Thus, the task of delineating “desirable” and “undesirable” motivations for animals’ participation in research is not straightforward.

Putting this issue to one side, it is worth pointing out that in some publications we surveyed, the entire purpose of the study was to understand what kinds of activities offer animals some benefit beyond just food. For example, this was the case in work aimed at improving animal husbandry, and in particular cognitive studies conducted in zoos (especially with primates; Table 1) where a goal was to test whether cognitive research tasks serve as enrichment for the animals (e.g., Herrelko et al., 2012; Ross, 2010; Ruby & Buchanan-Smith, 2015). In such studies, the reason that animals volunteer is crucial, not necessarily for determining whether a study counts as “voluntary,” but because the central goal is undermined if animals engage for the “wrong” reasons. For example, Washburn and Rumbaugh (1992) found that when given the choice between a cognitive task and “free food,” rhesus macaques more often chose the food. However, this result changed when the free food selection came with a 30-minute period when they could not access any cognitive tasks. As Ross (2010) summarizes, the result suggested that “there is ‘benefit’ to engaging in the tasks themselves above and beyond the value of the food alone” (p. 314) and therefore that this task acted as a genuine form of enrichment (see also Reinhardt, 1994). Animal studies like this perhaps come closest to the ideal of volunteering as involving no or minimal payment or other reward.

## What Does the Concept of Volunteering do in Animal Research?

Having explored how animal science papers differentially construct the relationships between volunteering, animal agency, and incentives offered by researchers, we are now able to critically reflect on what the idea of volunteering “does” in the context of animal research. In other words, if it is not operating as a distinctive and clearly defined scientific category, what role does it perform? We have identified at least three sets of functions.

First, by using language of agency and choice-making, one could argue that those working in animal research are making an important ethical intervention by implicitly acknowledging animals as agents − arguably, it has a similar effect to describing research animals as “working subjects.” However, in some publications we surveyed, the idea of animals as agents capable of feelings, wants, and intentions is not the general assumption. For example, studies of “voluntary exercise” in rodents (Table 1) have taken place since the late 19th century, often focusing on questions surrounding behavior motivation, with proposals ranging from hormonal status to food deprivation, play, stereotypic behavior, and genetics (see Greenwood & Fleshner, 2019; Kelly & Pomp, 2013; Sherwin, 1998 for reviews). It would therefore not be contradictory to do “voluntary exercise” studies with rats and believe that rats are essentially machines governed by their genes. Thus, in some cases the language of volunteering may be used without assuming anything about animal agency; rather, the term “voluntary” is shorthand for signaling that animals were given free access to a research activity, and that no physical force was involved. In such cases, animals’ voluntary action is viewed in purely physical terms, without reference to factors that are implied when the language of volunteering is applied to humans, such as mental states, contextual factors that constrain and shape choices, and comprehension of the harms and benefits of research. Arguably, this discrepancy reflects the legacy of dualist thinking, in which only humans are regarded as possessing a “mind” or “soul” while “animality” is conceived in largely physical terms (Midgley, 1978). Nevertheless, in other cases, use of the term “voluntary” does seem intended to imply animal agency. For example, studies aimed at offering cognitive enrichment for captive primates (e.g., see Herrelko et al., 2012; Ross, 2010; Ruby & Buchanan-Smith, 2015; Washburn & Rumbaugh, 1992) are premised on assumptions of primate intelligence, preferences, and feelings.

Second, using the term “voluntary” may also (intentionally or otherwise) frame research in a more positive light than if other terms, such as “work,” were used instead, given the more positive connotations of volunteering. The importance of this distinction is assumed in some human-animal studies literature that describes animal “work” as “grounded in constraint, suffering, and dependence” on humans (Porcher, 2017, p. 309; see also Barua, 2019; though see Coulter, 2016, for a broader definition of animal work that includes “voluntary labor” such as care work in human homes). Some animal researchers have attempted to draw similar distinctions. For example, Margulis (2017) makes the case that zoos need to “emphasiz[e] the differences between the observational, noninvasive, opportunistic, voluntary research conducted in zoos, and the sometimes invasive experimental studies that are often assumed to be the essence of any animal-based research” (p. 73). In other words, Margulis (2017) argues that describing zoo-based research as voluntary can help make it apparent that this activity is different (and, implicitly, more ethically acceptable) than much lab-based research.

Third, the language of volunteering is used by some authors to demonstrate their stated commitment to animal welfare, on the grounds that research described as voluntary is better for animal welfare than non-voluntary research. For example, Margulis (2017) highlights how voluntary research in zoos is part of a wider aim to use training to facilitate voluntary husbandry behavior, such as cooperating with medical procedures (Table 1; see Bloomsmith et al., 1998; Laule & Whittaker, 2007). In laboratories too, training is often used to facilitate voluntary husbandry, not only as a way of getting better research results (Schapiro et al., 2005) but also because of the positive welfare effects of “voluntary cooperation,” such as reduction in stress symptoms (e.g., Laule et al., 2003; Laule & Whittaker, 2007). Animal welfare is also a goal of many studies focused on providing cognitive enrichment. Nevertheless, it is important to remain critical of the welfare implications of “voluntary” activities, since they might also have negative effects − as Herrelko et al., (2012) states, “preference alone is not sufficient, as activities may have a detrimental impact” (p. 829; see also Neal Webb et al., 2019; Ross, 2010). Nevertheless, we maintain that at least some of the authors whose work we explored would appear to use the language of volunteering as part of an underlying ethical goal of improving animal welfare.

## Conclusion

This paper has shown that the language of volunteering is evident in different kinds of animal research literature, and that this language is utilized in several ways. While at first any reference to volunteering might be assumed to imply free, unconstrained, and unpaid participation in an activity, in reality, various human-imposed constraints are at play for the animal “volunteers.” These constraints can shape animals’ preferences and subjectivities before research takes place via captivity, breeding, and training, and constrain choice during research via the restriction of other options and use of rewards such as food.

In animal research, the extent to which these constraints are discussed or accounted for is highly variable. In some cases, the use of breeding, training, rewards, and restriction of alternatives are presented as compatible with “voluntary” behavior, implying that volunteering equates to an absence of physical force. However, in other cases, such as some primate cognition research in zoos, a deeper meaning of “voluntary” is implied, since the purpose of the research is often to offer choice and stimulation as methods for improving animal welfare. Indeed, some authors regard the use of training and rewards as incompatible with truly voluntary behavior (Chertoff et al., 2018).

Given this wide variation of use, we consider that it may be helpful for researchers to expand on what they mean by the term “voluntary” in writing about their study design, for example, by describing how factors such as breeding, training, individual personality, life history, and choice feature in their research. Though this would not lead to a single, consistent definition of voluntary animal research, it would at least enable readers to reflect on the appropriateness (or otherwise) of how the term is being used in a particular study.

In conclusion, it remains questionable whether “pure” voluntary behavior is ever attainable in research involving either human or animal subjects. Human agency is expressed relationally and within specific social contexts, and even apparently voluntary participation in human clinical trials may be shaped and constrained by specific social circumstances. In animals, “pure” voluntary behavior is perhaps even less likely, given the extensive constraints imposed by humans on research animals, the likely impossibility of accurately communicating to animals the justifications for research, the extent of harms they may experience while participating, and the difficulties involved in interpreting animals’ motivations. Yet the effort by some animal behavioral researchers to make animals’ participation as voluntary as possible, partly to enhance animal welfare, echoes Despret’s (2004, 2016) encouragement to work with animals in ways that seek to acknowledge animal agency, even if we are unable to secure informed consent. In other words, even if “pure” voluntary animal research is difficult to achieve, it is nonetheless a worthy aspiration, and one to be encouraged within animal research communities and beyond.

## Figures and Tables

**Table 1:** Common uses of “voluntary” in animal research literature

Use	Research areas	Brief description	Example literature extracts (underline emphasis added)
1 Body function	Evolution, neuroscience research	Describes certain body functions such as movements as voluntary, as opposed to involuntary, e.g., in discussions of evolutionary adaptations (Clark 2018) and neuroscience (Prochazka et al. 2000).	“If the sound is produced only in certain contexts …, it could be involuntary. Alternately, it could be under voluntary control of a ‘hidden switch’.” (Clark 2018)
2 Behavior in nature	Evolution, ethology, natural history research	In wildlife studies, terms may be used such as voluntary dispersal and voluntary avoidance (moving away from others: Murphy et al., 2018). “Willing” is also used, e.g., with reference to animals willing to take risks for mating and resources.	‘…subordinate males voluntarily disperse from their natal group to look for receptive females but can return to their natal group, whereas subordinate females rarely voluntarily disperse from their natal group.’ (Dantzer et al. 2019)
3 Medical procedures and handling	Often part of care rather than experiment, or research with an animal husbandry goal	Animals are described as voluntarily undertaking medical procedures like venipuncture, providing feces samples, or accepting restraint for medical or research procedures.	“… animals were trained by operant conditioning using food and verbal encouragements to allow voluntary physical examination …” (Kaartinen et al. 2018)
4 Participation in cognitive enrichment task	Psychology and cognition research, potentially with an animal husbandry goal	Usually involves giving free access to a test apparatus or testing area for cognition research. Task is usually assumed to be enriching/positive for the animal. Particularly common in zoos and with non-human primates.	“In most experimental studies of animal cognition, researchers attempt to control for multiple interacting variables by training subjects prior to actual testing, allowing subjects to participate voluntarily (‘free-choice’ participation), and/or providing subjects with food rewards to encourage their participation, motivation, and attention.” (Morton et al., 2013, p. 678)
5 Participation in aversive motivational test	Psychology research, especially on motivation and avoidance	Describes participation in more aversive procedures, e.g., mice crossing a mildly electrified grid voluntarily (Walker and Mason 2018). At other times, animals are described as “willing” to undertake an aversive task for a reward (e.g., Dunlop et al., 2006).	“This experiment was designed to allow mice to cross an electric grid *entirely voluntarily*. They did so in order to access an enriched cage, but both the starting cage and the enriched cage contained food, water, and nesting material, meaning that the mice *never* had to cross the electric grid if they found it too aversive.” (Walker and Mason, 2018, p. 102)
6 Exercise	Research on motivation for exercise	“Voluntary exercise” is used when laboratory rodents are given free access to exercise wheels, and often contrasted with “forced treadmill training.”	“. with many studies utilizing ad libitum access to voluntary exercise wheels, while others employ comparably short bouts of forced exercise on a treadmill.” (Leasure and Jones, 2008, p. 456)
7 Movement between enclosures	Agricultural and zoo husbandry research	Refers to movement between areas, as in “voluntary traffic” in agricultural animals (Wildridge et al., 2018), or to movement between enclosures in zoo- or lab-housed animals.	“Positive reinforcement techniques were applied to train groups of chimpanzees to move voluntarily into the indoor portions of their enclosures at the request of trainers …” (Bloomsmith et al., 1998, p. 333)
8 Ingestion and consumption	Addiction studies, agricultural husbandry research	Used with reference to voluntary consumption of food in agricultural studies, or to voluntary consumption of drugs in addiction studies (Uhart and Wand 2009). Term “voluntary” may also be used with reference to refusal to eat (Grant et al. 2018).	“The amount eaten during a period of time (usually 1 d) is usually called the *voluntary intake;* this is often lower than the *potential intake* …” (Forbes, 2013, p. 5)
9 Interaction with humans	Agricultural or companion animal husbandry research	Often with reference to interactions with a caregiver, as in the “voluntary human approach test” (which may be contrasted with the “forced human approach test”).	“The exposure to a human has been specifically devel oped for farm animals to study their fear-related responses, with the animal that is either approached by a human (‘Forced Approach test’, FAT) or is free to approach a human (‘Voluntary Approach test’, VAT).” (Forkman et al., 2007, p. 341)
